# Observation and Measurement of Ice Morphology in Foods: A Review

**DOI:** 10.3390/foods12213987

**Published:** 2023-10-31

**Authors:** Indira Pérez-Bermúdez, Alison Castillo-Suero, Anielka Cortés-Inostroza, Cristóbal Jeldrez, Adriana Dantas, Eduardo Hernández, Patricio Orellana-Palma, Guillermo Petzold

**Affiliations:** 1 Grupo de Crioconcentración de Alimentos y Procesos Relacionados, Departamento de Ingeniería en Alimentos, Facultad de Ciencias de la Salud y de los Alimentos, Campus Fernando May, Universidad del Bío-Bío, Av. Andrés Bello 720, Chillán 3780000, Chile; 2 Departamento de Ingeniería en Alimentos, Facultad de Ingeniería, Campus Andrés Bello, Universidad de La Serena, Av. Raúl Bitrán 1305, La Serena 1720010, Chile; alison.castillos@userena.cl (A.C.-S.); anielka.cortes@userena.cl (A.C.-I.); cristobal.jeldrez@userena.cl (C.J.); 3 Institute of Agrifood Research and Technology (IRTA), Food Quality and Technology, Finca Camps i Armet, Monells, 17121 Girona, Spain; adriana.dantas@irta.cat; 4 Agri-Food Engineering and Biotechnology Department, Campus del Baix Llobregat, Universitat Politècnica de Catalunya BarcelonaTech, Edifici D-4 C/Esteve Terradas, 8, Castelldefels, 08860 Barcelona, Spain; eduard.hernandez@upc.edu

**Keywords:** microscopy, microstructure, morphology, ice crystal, freezing

## Abstract

Freezing is an effective technology with which to maintain food quality. However, the formation of ice crystals during this process can cause damage to the cellular structure, leading to food deterioration. A good understanding of the relationship between food microstructure and ice morphology, as well as the ability to effectively measure and control ice crystals, is very useful to achieve high-quality frozen foods. Hence, a brief discussion is presented on the fundamentals/principles of optical microscopic techniques (light microscopy), electronic microscopic techniques (transmission electron microscopy (TEM) and scanning electron microscopy (SEM)), as well as other non-invasive techniques (X-rays, spectroscopy, and magnetic resonance) and their application to measuring ice formation rates and characterizing ice crystals, providing insight into the freezing mechanisms as well as direct monitoring of the entire process. And, in addition, this review compares (the negative and positive aspects of) the use of simple and cheap but destructive technologies (optical microscopy) with detailed microscopic technologies at the micro/nanometer scale but with pretreatments that alter the original sample (SEM and TEM), and non-destructive technologies that do not require sample preparation but which have high acquisition and operational costs. Also included are images and examples which demonstrate how useful an analysis using these techniques can be.

## 1. Introduction

The study of food microstructure has become increasingly important for researchers, since it can have a clear influence on different nutritional values, rheological behaviors, textural characteristics, and sensorial qualities, as well providing information about precise use conditions in the application of traditional processes or modern innovative technologies [1]. In particular, the freezing process, a traditional food technology, has habitually been associated with the storage and preservation of foods, since water’s activity can be reduced by maintaining a low-temperature environment; thus, chemical and enzymatic reaction rates are decreased, preventing the growth of unwanted microorganisms and, in turn, allowing the extension of the shelf life of foods and an increase in the conservation of valuable nutritional and organoleptic attributes [2,3,4,5].

**Figure 1:**
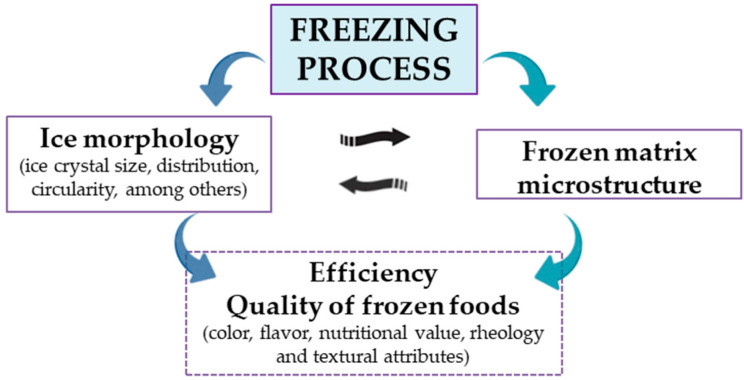
Most important parameters during the freezing process of foods.

Specifically, the crystallization of water during freezing is the most important step of the freezing process, since ice nucleation occurs and initial crystals begin to grow in a dispersed dendritic form in the supercooled water, ending the freezing process, with the growth of concentric rings of solid ice at 0 °C and the final cooling of the solid ice [6]. Thus, once the freezing process has finished, if the ice crystals are large, they cause damage to the food microstructure during the thawing process, resulting in the modification of food characteristics, such as nutrients, color, flavor, aroma, and texture, due to the high degree of tissue softening, increased drip losses, and reduced water-retention capacity and tenderness, and the darkening of tissues, among other characteristics [7]. Therefore, understanding the relationship between ice morphology and the frozen-matrix architecture, as well as changes in the components of the microstructure during the freezing process, are very useful to achieve high efficiency in the freezing process, leading to an improvement in the quality of frozen products (Figure 1) [8,9,10].

In practical terms, ice morphology (circularity, size distribution, clast roundness, fractal dimension, among others) is a relevant parameter during the freezing process of solutions and/or liquid foods [8], since it is closely related to the growth and formation of ice crystals (cooling temperature and heat transfer area), the crystal growth rate that affects ice nucleation and, later, ice crystals’ generation and the temperature and presence of solutes [11].

In this context, ice morphology plays a key role in the quality of frozen foods along the cold chain: (1) A rapid freezing rate is very important because it determines the ice morphology (size and shape of ice crystals) within a food product. In tissue foods, a slow rate of freezing causes an ice morphology with large ice crystals in extracellular locations, resulting in poor frozen food quality. In contrast, a quick freezing rate produces an ice morphology with small crystals at locations inside and outside of the cells, and, as a consequence, high-quality frozen foods. (2) The freezing temperature should be kept constant, because any fluctuations in the storage freezing temperature tend to reduce the quality of frozen foods; for example, drip losses after thawing, which are mainly due to ice morphology changes as a result of recrystallization [8].

Thus, techniques for the direct visualization of ice morphology have been developed through qualitative and quantitative measurements [12,13], contributing to our understanding of the structures of food materials [14]. As result, micro-X-ray-computed tomography (μCT), which has a minimal intervention in the samples, dynamic in situ microscopy, and a high appreciation of image analysis are increasingly used to obtain information from the microscopic images of frozen foods or samples undergoing the freezing process [15]. Thereby, some microscopy technologies have been successfully used for the observation of ice crystals in foods, such as optical microscopy (LM) [16], transmission electron microscopy (TEM) [17], scanning electron microscopy (SEM) [18], and confocal laser scanning microscopy (CLSM) [19]. Moreover, indirect or nondestructive electromagnetic spectroscopic techniques, such as nuclear magnetic resonance (NMR) and magnetic resonance imaging (MRI), can be used to analyze image-based ice crystals, taking into account the behavior of water [20]. Hence, the development and application of novel microscopic techniques for the visualization of ice crystals in food samples have been reported, for example, by Kono et al. [21], who presented some advances in ice crystal measurements through a microscope using fluorescence on the surface color of a model food. Later, the results were related to the freezing rate, ice crystal size, and color of the whitening of a salmon fillet surface during freezing, while Pu et al. [22] analyzed the microstructure of cake using X-rays, and Zhuang et al. [23] detected ice crystals in frozen pork using fluorescence hyperspectral imaging. Similarly, there are also excellent reviews of the material and structure of foods subject to low-temperature processes, and the application of microscopy techniques to visualize ice crystals that have provided a valuable introduction in relation to freezing technologies [24,25,26].

Based on the key role of ice morphology for the quality of frozen food products, it is necessary to have updated and easily accessible scientific information for researchers related to the different techniques available for the observation and measurement of ice morphology in different experimental conditions, the temperature and freezing technique applied, as well as different food matrices. Therefore, the aim of this review is to discuss the use of microscopy in the study of the ice morphology and microstructure of frozen foods, highlighting the effects of the different low-temperature processes on the food quality properties. A brief discussion of the fundamentals and principles of the different microscopic techniques (optical and electronic), as well as other non-invasive or destructive techniques (X-rays, spectroscopy, and magnetic resonance) is carried out. Additionally, examples of their application to the study of the changes in frozen foods’ structure are shown, as well as their effectiveness in measuring the ice formation rate and characterizing the crystals’ morphology, providing information on the freezing mechanisms and allowing for the constant monitoring of the process.

## 2. Methodology 

Our review was carried out using the keywords (microscopy, microstructure, morphology, ice crystal, and freezing) in the Scopus and ScienceDirect databases from 2008 to 2023.

Based on the >500 articles chosen from the Scopus and ScienceDirect databases, the paper selection was restricted to articles related to the different microscopic techniques used in ice crystal visualization such as optical microscopy (also called light microscopy, a microscopy technique that uses visible light with a lens system to generate images at different magnifications of small objects) [16], electron microscopy (using a beam of electrons as the source of illuminating radiation, with different electron microscope techniques, such as TEM, where a high energy electron beam is transmitted through an extremely thin sample, and SEM, where it produces images of a sample by scanning the surface with a focused beam of electrons) [17,18], and non-destructive and non-invasive microscopy techniques, i.e., without the need to cut the specimens (for example, X-ray CT, where it characterizes structures three-dimensionally, and thus the sample can be evaluated microstructurally at nanoscale resolutions; AFM, where the image is obtained by “touching” the surface of the sample; and Raman spectroscopy, where it provides a structural fingerprint through vibrational modes of molecules) [22].

Additionally, among all the articles, only 109 were related with microscopy techniques to analyze ice crystals in food matrices.

## 3. Optical Microscopy Applied to Frozen Foods

### 3.1. Light Microscopy (LM) and Cryo-Light Microscopy (Cryo-LM)

LM is commonly used to provide microscale evidence on the structure of different foods, since this direct observation technique has advantages that facilitate its use such as a modest start-up capital, low maintenance costs and convenience, full color information with high resolution in the images obtained (aiding both interpretation and visualization of food microstructure), and the samples can be totally wet and these can be analyzed under ambient conditions, but the LM technique presents disadvantages such as a limited depth of focus, difficulty removing the presence of dirt or foreign bodies that prevent the visualization of samples, magnification lenses are fixed (limiting the resolution in the images), some samples need to be treated with staining to produce specific coloration of particular substances, and in turn, some samples can only be observed with the implementation of a cryogenic device (Cryo-LM) [27]. Thereby, the study of the structure of frozen foods with LM equipment can be completed at low temperatures when the sample is frozen using a cold chamber or by using a cryogenic microscope [9]. Specifically, a Cryo-LM consists of a temperature control system or a heat transfer stage installed in the LM equipment. Thereby, the microscope can be equipped with a camera, and thus, the freezing process can be controlled to determine aspects such as the ice nucleation temperature, size of ice crystals, shape and location of ice crystals, mechanical effects of ice crystals on cells and tissues, and volumetric and morphological changes of cells [28]. However, the only real Cryo-LM limitation corresponds to a lower spatial resolution of visible light optically, but Cryo-LM is much faster than the LM technique, since the sample preparation stage is eliminated [29].

**Figure 2:**
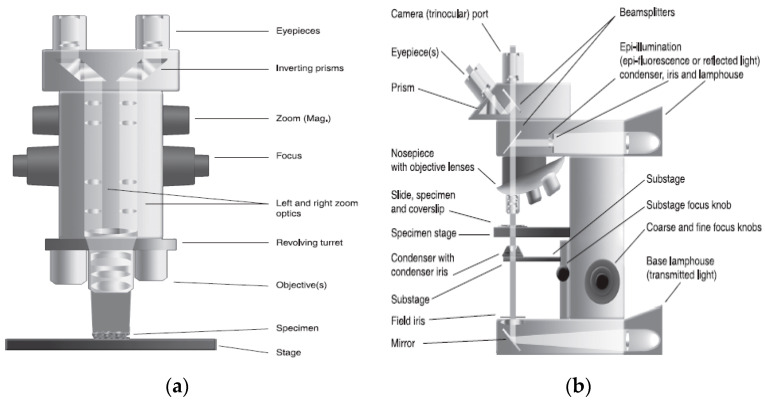
Diagram of optical microscopes: (**a**) Stereomicroscope; (**b**) Compound microscope [27] (with permission).

In particular, LM can be distinguished under two major classes (Figure 2), stereomicroscopes (Figure 2a) and compound microscopes (Figure 2b), where stereomicroscopes (an inspection microscope) present low to intermediate magnifications (between 5× and 200×), and the objectives can be changed to vary magnifications/resolving power through a turret/nosepiece, and thus, the images are obtained by two lights inclined at a small angle, while compound microscopes allow one to multiply the magnification of the objective by one or several additional lenses, including the eyepiece lens. This microscope achieves magnifications between 10× and 1000×. Its main advantage corresponds to a higher resolution performance, as working distances are reduced (one to two hundred micrometers) [27]. 

### 3.2. Application of LM and Cryo-LM to Visualizate Ice Crystals in Frozen Foods

Thus, these microscopy techniques (LM and Cryo-LM) allow the observation of changes in ice crystals during the freeze-thaw process, presenting the advantage that the sample preparation is not complicated, and thus, LM and Cryo-LM provide microscale data on frozen food structures and lyophilized and thawed samples [11]. Hence, LM and Cryo-LM have been used to study the microstructure of different food products. For example, Moreno et al. [30] studied an emerging nonthermal technology called freeze concentration in coffee extract in terms of volatile compounds, sensory quality, and ice morphology, and the results showed that the ice crystal sizes were lower for the falling-film freeze concentration (from 0.008 to 0.020 mm^2^ and 0.065 to 0.100 mm, for area and diameter, respectively) than block freeze concentration (from 0.008 to 0.080 mm^2^ and 0.064 to 0.196 mm, for area and diameter, respectively), but in all cases, the ice crystals presented a shape of solid prisms or plate ice morphologies [31]. Likewise, Wang et al. [32] focused on the quality changes with the ice crystals’ formation during frozen storage in the dorsal ordinary muscle of horse mackerel (*Trachurus japonicus*). The histological structure results indicated damage by numerous intracellular and extracellular ice crystals (between 50 to 100 μm) in muscle cells of frozen muscle specimens and small interstices between cells of freeze-thawed muscle specimens, i.e., there is no presence of ice crystals after thawing process. Moreover, Du et al. [33] studied the cryoprotective effect of ice structuring protein (ISP) in mirror carp during various freeze-thaw processes, where the ice crystals (observed by LM technique) showed an average diameter of 220 μm without ISP after five freeze-thaw processes, while with ISP, the average diameter of ice crystals was reduced by 13.6% after the same freeze-thaw processes, and actually, Li et al. [34] studied the effect of inhibition of cellulose nanocrystals at different concentrations on ice recrystallization in sucrose solution, and the results obtained by a polarized light microscopy showed that high cellulose nanocrystals concentration inhibited the ice recrystallization, relating with the accretion of nearby ice crystals and large ice crystals’ formation with irregular morphology and high aspect ratios, with values between 38 to 42 μm, 65 to 85 μm, and 50 to 70 μm, at 3.0%, 25.0%, and 40.0% of sucrose solution, respectively.

Thus, Table 1 summarizes the application of LM and Cryo-LM techniques for the observation and measurement of ice crystals in various frozen food samples.

## 4. Electron Microscopy Applied to Frozen Foods

In the last 40 years, electron microscopes have emerged as a powerful tool for the characterization of a wide range of materials, since these microscopes present a high versatility and extremely high spatial resolution, and thus, electron microscopy is a valuable tool for many applications, including food areas, where the understanding of structure in various foods has improved greatly, and thus, new foods have been developed [49]. In particular, electron microscopes are based on the imaging principle in a similar way to optical microscopy, with the difference that, in this case, the illumination source is a beam of electrons, and this is the motif that the electron microscope resolution is almost 5 nm, while in the optical microscope, the resolution is only 200 nm [50].

Specifically, electron microscopy corresponds to different techniques, and each technique has been used with great success for freezing-related evaluations, including transmission electron microscopy (TEM), scanning electron microscopy (SEM), cryo-SEM, and environmental scanning electron microscopy (ESEM).

### 4.1. TEM

In TEM, it is possible to obtain refined internal information from the sample as they are penetrated by short wavelength electron beams and form a highly magnified and detailed image in a vacuum environment. Hence, it provides powerful magnification and valuable information on surface, shape, size, and structure features [50].

Thereby, TEM is used to analyze the effects of ice crystals on the internal structure of frozen foods instead of trying to observe them directly; for example, Zhang et al. [51] investigated the effects of multiple freeze-thaw (F-T) cycles on water mobility, microstructure damage, and protein structure changes in porcine longissimus muscle. Repetitive F-T cycles led to changes in the secondary and tertiary structures of the myofibrillar protein, and in turn, the formation of large ice crystals in various F-T cycles had a significant change in the myofibrils, since the myofibrils presented a significant shrinkage, leading to a remarkable separation between myofibrils.

**Figure 3:**
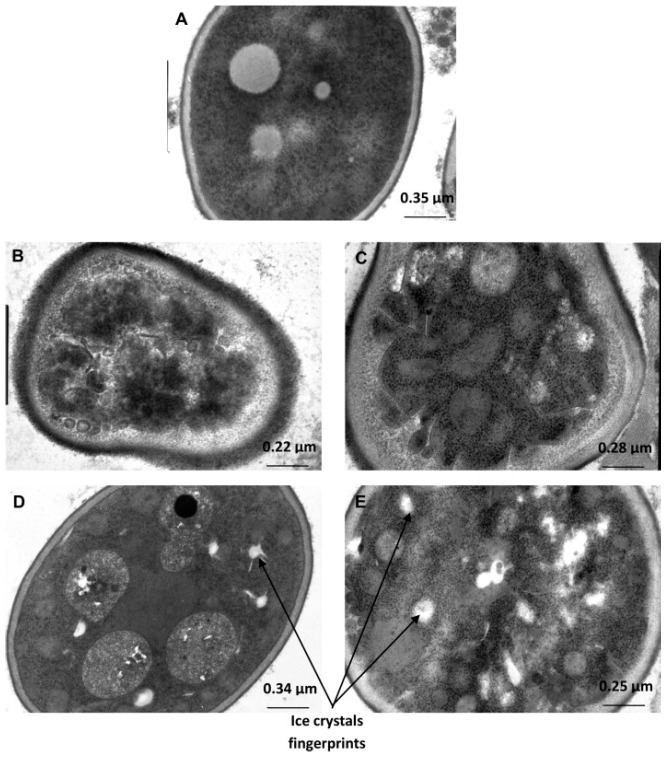
TEM observation of dough under different freezing treatments: (**A**) Unfrozen dough; (**B**) freezing at −20 °C; (**C**) freezing at −30 °C; (**D**) freezing at −40 °C; (**E**) freezing in liquid nitrogen [52] (with permission).

In another TEM observation, Meziani et al. [52] studied the effect of freezing treatments (−20 °C, −30 °C, −40 °C, and liquid nitrogen) on fresh and thawed sweet doughs. The results indicated an excellent network integrity depend on the freezing rate, since the different temperatures control the size and location of the ice crystals. Specifically, small ice crystals were unstable, and thus, the crystals could recrystallize into large crystals during thawing, causing significant damage to the cells inside the sweet doughs (Figure 3).

However, this technique had some limitations, since the observed sample must be very thin (generally less than 150 nm) because the electron beam can be easily scattered, and in addition, the sample preparation is quite a complex and tedious procedure. Furthermore, the samples are limited to those that are electron transparent, capable of tolerating the vacuum chamber, and small enough to fit in the chamber [11].

### 4.2. SEM

SEM obtains images through electron high-energy beam scanning, magnifying by using electrons, allowing a wide range of magnifications, and it can achieve a depth of field approximately 500 times greater with the object magnification from 10 to 300,000 times. Thereby, SEM has been a very attractive and powerful electron microscope for food scientists, since it allows the study of the internal and surface characteristics of food materials. Thus, some advantages include a simple sample preparation, a wide magnification range, a large depth of field, and the images are a representation of electronic data, and hence, it allows a better analysis and quantification of images. In addition, it combines the characteristics of optical microscopy and TEM. However, a clear disadvantage is the differentiation of agglomerated structures, i.e., SEM (and image resolution) depends on the interaction between the sample and electron beam, but an important point, the agglomeration of nanoparticles and their particular differentiation in a sample are not clearly appreciated and total dehydration is required for biological materials [53].

**Figure 4:**
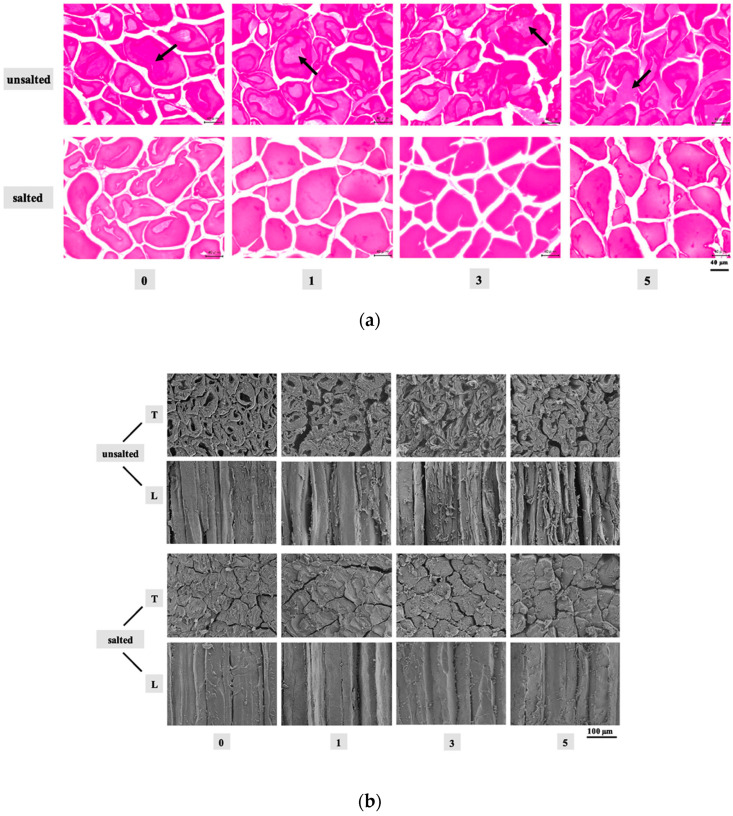
Tuna meat subjected to different freeze-thaw cycles, where 0, 1, 3, and 5 correspond to the number of freeze-thaw cycles, and arrows indicate cellular substances: (**a**) Cross-sectional LM technique, where arrows indicate cellular substances; (**b**) SEM images, where T and L correspond to the transverse direction and longitudinal direction, respectively [55] (with permission).

Specifically, Ban et al. [54] used SEM to study the internal structure of croissant dough during the cooling process and to evaluate the effects of controlled freezing on croissant quality. In this study, the images obtained from the doughs showed a typical structure that includes the gluten networks, voids, and starch granules, and angular voids were detected that represent the ice crystal spaces, coinciding with their final size. As the freezing speed increased, the size of the crystals decreased, being an important factor, since the ice crystals affect the viability of the yeast, influencing the specific volume and the firmness of the croissant. Therefore, the freezing speed and temperature applied to the croissant dough must be kept above the crystallization point of the yeast cytoplasmic water with optimal values of −3.19 °C/min and −20 °C, respectively. In the same way, SEM has also been very useful in combination with other microscopy techniques to assess ice morphology and frozen cell tissues. Jiang et al. [55] investigated the influence of freeze-thaw cycles on the stability and morphological properties of salted and unsalted tuna meat tissues (Figure 4), where a cross-sectional LM technique in the samples without salt indicated that, as the freeze-thaw cycles increase, large crystals are formed extracellularly and intracellularly, while in the salted samples, large crystals are formed intracellularly. By SEM, it was observed that the ice crystals in the salted samples are spherical, while in the unsalted ones, the ice crystals formed elongated columns attributed to the solubilization of the myofibrillar proteins. These elongated crystals in the samples without salt cause high stress to the thawed cellular material, and the ability to recover the tissue microstructure decreases after the thawing step.

#### *Cryo*-*SEM*

Another method used for the evaluation of frozen and freeze-dried foods is cryo-SEM. With a special cooling system, cryo-SEM supports the observation of frozen samples, allowing direct observation without a thawing step. Additionally, it provides an interesting alternative, since the unfrozen samples can be placed under the SEM equipment and can be gradually frozen, and thus, the sample is frozen and kept in the microscope at low temperature, allowing the investigation of the internal three-dimensional structure of wet samples. Cryo-SEM provides rapid physical fixation, avoiding the risk that other artifacts can be added in the preparation procedure, leading to chemical fixation, structural collapse, or cell contraction as sometimes occurs in typical preparation methods. However, as well as the SEM method, the main drawback corresponds to its high cost of acquisition and maintenance of instruments [56]. This is a clear utility of cryo-SEM. Sriamornsak et al. [57] used cryo-SEM in calcium pectinate gel (CaPG) beads, since SEM did not allow the in situ observation of hydrated beads, i.e., it only allowed observation of the bead structures subjected to freeze-drying technology.

In food terms, cryo-SEM allows the ice crystals’ investigation in hairtail samples under three different frozen treatments, where the results showed ice crystals with an equivalent diameter close to 12.6 μm, 3.6 μm, and 1.2 μm for −20 °C, −80 °C, and −196 °C, respectively, with more small and regular ice crystals in samples frozen at −196 C in relation to the other freezing treatments [58]. Moreover, Kong et al. [59] studied the effects of different antifreeze peptides as a pretreatment on frozen cherries, evaluating their potential to minimize freeze-thaw damage and preserve structure, texture, and nutritional values. The results (at 50 μm) indicated that large ice crystals of abnormal shape caused cell wall collapse in control frozen cherries (untreated with antifreeze peptides), while samples with antifreeze peptides showed a compact and stable structure due to the formation of small ice crystals during freezing, minimizing damage to the cell membrane and cell wall.

**Figure 5:**
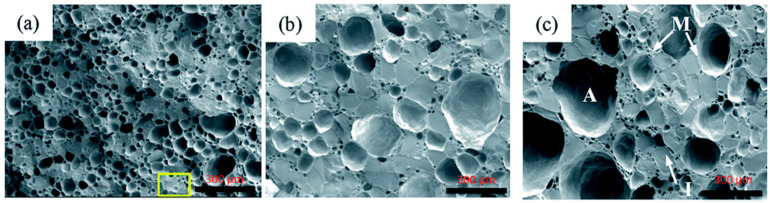
Microstructure of ice cream by cryo-SEM: (**a**) Initial ice cream; (**b**) ice crystals after 7 freeze-thaw cycles between −5 °C and −15 °C; (**c**) ice crystals after 14 freeze-thaw cycles between −5 °C and −15 °C [60] (with permission). The yellow rectangle in (**a**) correspond to enlarged region from 300 mm to 40 mm (figure not shown). The letters A, I, and M in (**c**) stand for the air cell, ice crystal, and unfrozen matrix, respectively. Scale bar is 300 mm.

Furthermore, Guo et al. [60] analyzed the microstructure of the ice cream using cryo-SEM after 0, 7, and 14 freeze-thaw cycles between −5 °C and −15 °C. In the fresh sample, the ice crystals had an uniform dispersion and size close to 34 μm. After the seventh freeze-thaw cycle, the ice crystals had a statistically similar size (equivalent diameter) ≈68 μm, but the total number was less than the fresh sample. The images indicated a clear connectivity of air cells and ice crystals in the fresh sample, but the connectivity (less thick walls) was affected as the cycles progressed (Figure 5).

### 4.3. ESEM

Due to the SEM limitations (it operates under high vacuum conditions, requiring the operation of the electron gun), ESEM was established to enhance the ice crystals’ visualization, and thus, ESEM differs from conventional SEM mainly by the presence of a gas in the sample chamber. The presence of a gas or environment around the sample is the inspiration for the term ‘‘environmental’’, and it can play two main roles [61]. In the first role, the gas acts as an electrical charge conductor, avoiding that the sample charging, and thus, it facilitates signal detection. The second role, more specific to ESEMs, is thermodynamic, i.e., the gas is a medium that prevents the evaporation of liquids from a sample [62].

In a recently published review, Pach and Verdagues [61] explain the ESEM use to understand the ice nucleation and the authors propose the novel uses that ESEM can offer for future food science and engineering investigations. For example, Hajji et al. [63] studied the effects of the dehydrofreezing on the microstructure of strawberries, where the images showed ice crystals with a large and irregular shape, leading to the tearing of structures due to the volumetric expansion which resulted from the crystallization of water, causing irreversible mechanical injuries.

Thereby, the use of optical and electron microscopy, as well as other techniques for the measurement, evaluation, and control of ice crystallization, and analysis of the characteristics of ice crystals before, during, or after the freezing process, has been addressed by many authors, with the aim of understanding the relationship between microstructure and ice crystals in food quality. Additionally, the different investigations discussed novel technologies for the control of the ice crystals’ formation based on ultrasound, high pressures, electromagnetic fields, and the use of biological proteins by inducing or suppressing crystallization, to assist or accelerate the processes of freezing, showing great potential in obtaining a higher quality in frozen foods with smaller and evenly distributed ice crystals.

Hence, Table 2 summarizes the application of electron microscopies for the observation and measurement of ice crystals in various frozen food samples.

## 5. Other Microscopy Techniques

As previously mentioned, optical and electron microscopies are widely used visual technologies for measurement and characterization of ice crystals and ice formation rate, as well as monitoring the entire freezing process. Hence, these technologies have also been used to perform a structural examination of frozen foods, including evaluating microstructural damage to cells and tissues, observing solute redistribution, establishing the degree of food heterogeneity, and important correlations between the microstructure and the texture of the mouth [53]. Nonetheless, optical and electron microscopies have limitations. For example, the resolution and magnification of the LM equipment, on some occasions, do not allow for a clear image. SEM and TEM require vacuum pretreatment and/or optimal staining and sample storage time for visualization, and it can sometimes cause changes in the initial morphology of the sample. Hence, atomic force microscopy (AFM) with an atomic resolution microscope has been studied in ice crystals, since AFM does not require sample coating and AFM operates in an atmospheric or liquid environment, but it has the disadvantage that the range and image speed are limited [11]. Thereby, to increase the measurement precision and to avoid the damage produced in the samples due to the preparation methods, non-destructive and non-invasive techniques have emerged based on different radiation ranges such as: X-ray computed tomography (X-CT) [74], near infrared spectroscopy (NIR) [75,76], Raman spectroscopy [77], magnetic resonance imaging (MRI) [78], and nuclear magnetic resonance (NMR) [79]. Hence, these techniques allow the transformation of absorbed signals (X-ray, NIR, and NMR) or scattering (Raman spectroscopy) into electrical signals, obtaining internal information of the samples [80].

### Application in Frozen Foods

**Figure 6:**
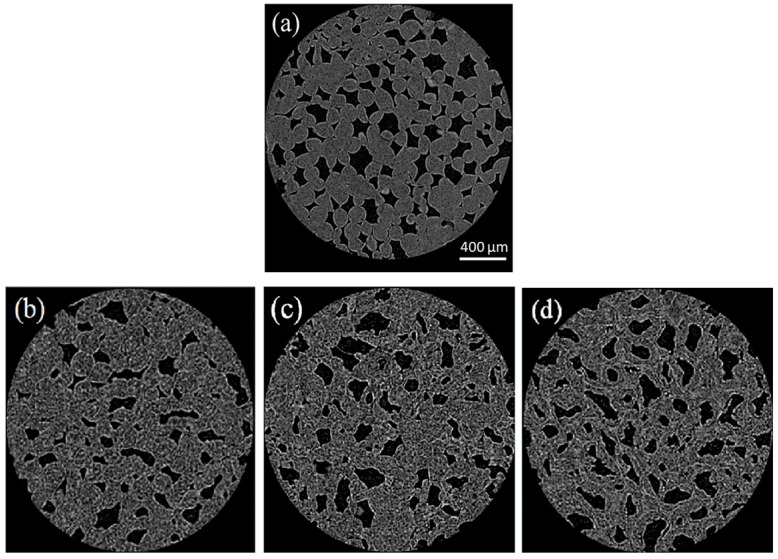
μCT of slices of apple samples subject to the different freezing conditions using a Skyscan 1172 μCT system at a 3.8 μm pixel resolution: (**a**) Fresh apple tissue sample (dark regions represent pores and gray regions correspond to cells); (**b**) frozen apple sample subjected to fast freezing; (**c**) intermediate freezing; (**d**) slow freezing (dark regions represent the pores, gray intermediate regions correspond to the ice crystals, and light gray pixels denote the matrix not frozen by the different methods) [85] (with permission).

In the same way, computed tomography and infrared (IR) analysis have been used to study the freezing process and to monitor the state of water during freezing. IR spectroscopy has proven to be a good and reliable method for detecting ice nucleation and for studying crystal growth because the IR absorption spectra of ice and liquid water are very different. These techniques, being non-invasive methods, do not introduce processing artifacts and allow repeated observation of the same sample as it undergoes controlled changes during processing [75]. Meanwhile, X-ray CT has been a powerful tool for evaluating and visualizing the three-dimensional (3D) microstructure of different frozen foods such as beef [76], fruits [81], vegetables [82], tuna meat [83], and sponge cake [84]. In particular, Vicent et al. [85] used X-ray micro-CT in development and validation to visualize, characterize, and quantify the 3D microstructure and ice crystal size distribution in frozen apples at three different freezing rates. The results presented interesting effects on frozen tissue, producing different sizes of ice crystals and an increase in cell diameter relative to fresh apples (Figure 6). Quantitatively, the authors concluded that the patterns of the ice crystals define the evolution of the tissue microstructure, where larger ice crystals cause greater damage to the structure, and therefore, an undesirable effect on the fruit quality.

**Figure 7:**
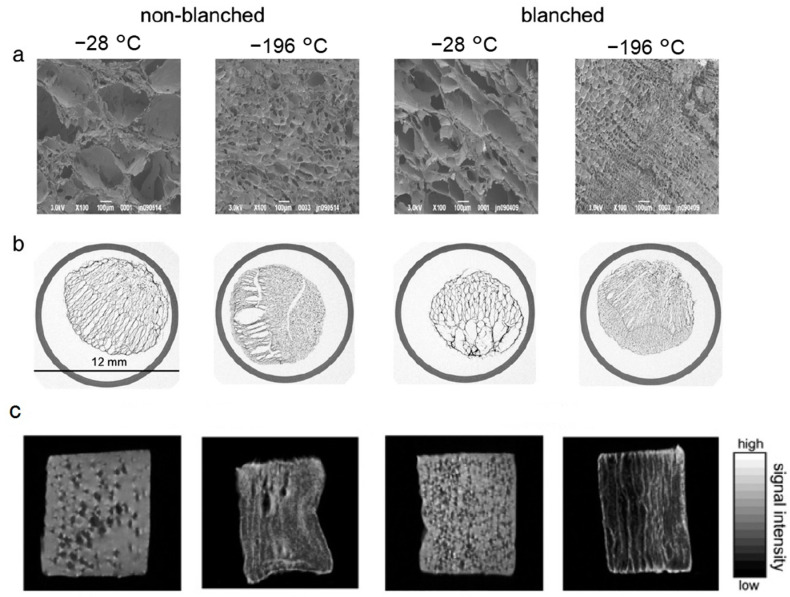
Images of the lyophilized carrot tissue at −28 and −196 °C with and without blanching treatment: (**a**) SEM; (**b**) μCT; (**c**) MRI [20] (with permission).

Another example is the study reported by Zhao and Takhar [86], the 3D ice crystal morphology, size distribution, and volume fraction in frozen potatoes was quantified by X-ray microCT under simulated temperature fluctuations during industrial storage and shipping. The results can be used to model and design transport phenomena of freeze-thaw processes, and in turn, to improve and to understand the texture attributes of frozen products. Moreover, the impact of thermal pretreatment and freeze-drying on the microstructure and rehydration of carrots was investigated by Voda et al. [20] with the combination of μCT and SEM techniques to visualize the microstructure at the μm-mm level by comparing the pore size obtained by image analysis of ice crystals induced by dendritic growth (Figure 7), and in addition, MRI and NMR were used to evaluate in a non-invasive way the mobility of water in the rehydrated samples and the integrity of the tissue compartment and the permeability of the plant material. Specifically, the dry microstructure state and the integrity of cell tissue integrity obtained by SEM can be observed (Figure 7a), where the sublimation of the ice crystals grown within the carrot leaves form a dry matrix that represents a fingerprint of the sizes and shapes of ice crystals. Frozen samples at lower temperatures showed low pore sizes because the ice crystals grew less under rapid cooling conditions. However, the growth of the ice crystals broke, pushed, and compressed the cells during the freezing process. This damage is more pronounced in slowly frozen tissue, where larger ice crystals were obtained. Blanched samples showed a finer structure and a better preservation of the microstructure with the increase in freezing speed in combination with blanching. Meanwhile, for μCT images (Figure 7b), from a qualitative perspective, the images reveal a dry matrix composed of pores and cavities that remain after the ice sublimation in the carrot tissue. The direction of the ice crystals’ formation can be clearly seen in the slowly frozen samples. In relation to the microstructure, slow freezing causes the crystals to grow out of the cell causing damage by its collapse and rupture, while during rapid freezing, the crystals grow within the cell with less separation between them and much less cellular damage. With MRI, the water distribution can be observed in rehydrated samples (Figure 7c), where artifacts appear with the samples due to the presence of air in the non-hydrated pores. The intensity of the signal depends on the amount of water and the density of the tissue by pixel. The difference in the structure between the frozen samples slowly with and without blanching can be identified by the small non-hydrated cavities present in the blanched samples. In addition, the distribution of water in these samples rehydrated at -196 °C indicates a microstructure similar to the structure of native carrot.

In summary, the techniques used to observe and measure ice morphology have been summarized in Table 3.

## 6. Conclusions and Future Challenges

Freezing has achieved resounding success in relation to food preservation, since it manages to preserve the nutritional elements of different foods. The types of frozen foods range from raw commodities such as fruits, vegetables, and meats to processed foods such as dairy desserts and frozen dinners. The structure of the ice phase in frozen foods is important at both the microscopic and macroscopic levels. The size and number of ice crystals determine sensory properties such as ‘hardness’ or ‘iciness’ in foods that are consumed without thawing, but they cause negative changes in many foods that are perceived in food quality when it is thawing. Additionally, a good control of the freezing rate parameter allows a high frozen food quality, since this parameter determines the size and shape of ice crystals, including mechanical stresses in the frozen foods, where, as the theory mentions, a fast freezing rate generates a small ice crystal size, while a slow freezing rate generates a large ice crystal size. Thus, a food with a cellular structure can be affected by the ice crystal size, since the cellular structure/ice crystal size ratio is a critical measure with respect to the texture and water holding capability, among other food characteristics. Hence, large ice crystals puncture the cellular structure (cell membranes), provoking loss of the intracellular fluid during thawing, and thus, the texture is unappetizing and mushy (for example, in meat and fish). Meanwhile, in ice cream, small round ice crystals allow a soft and creamy texture. Therefore, depending on the food, the freezing rate parameter can have different (positive and negative) effects on the (micro)structure of the food due to the formation of ice crystals.

The purpose of this review was to show an overview of applications related to different microscopic observation techniques and the different uses in the frozen food industry. With the development and availability of microscopy, spectroscopy, and computer vision, new methods have appeared for the acquisition, direct monitoring, and study of ice crystals, microscopic frozen food structures, and associated processes. 

Thereby, as stereomicroscopes and compound microscopes present a low/regular resolution and the sample can be destroyed by the preparation procedure, novel optical and electron microscopies have been developed to obtain more complete sample information. Furthermore, spectral imaging and online techniques have shown auspicious results on the ice crystal behavior. More importantly, the emergence of the latter makes in situ detection a reality. However, the most appropriate technique to be used will depend on the sample property of the studied sample and its nature. This review shows that these imaging techniques can be used to find the relationships between food processing conditions and the morphological changes of its components, allowing higher quality products and improving associated processes. However, there are two important limitations in some of these methods, mainly they require special equipment (cryo-section) or cooled sample holders (Peltier) to keep the sample at temperatures below the freezing point during the measurement, as well as the availability of a cryostat to cut the sections of the frozen samples. Some of these innovative technologies are in development or at the laboratory stage, while for others the biggest obstacle is the high capital cost.

Nevertheless, as these technologies provide sample images at different angles, more and more combinations will appear from these observation techniques which will allow more information for a better understanding of ice formation mechanisms. For this reason, non-destructive and online measurement will provide real information of the studied sample and will be an inevitable trend in the future. These non-destructive and online techniques have great future potential, since they allow costs to be reduced by keeping food samples unchanged when tested for their ice morphology in a production line using computational simulation models and automatic control systems. On the other hand, an important challenge is to couple the control parameters with artificial intelligence that allows prediction of the final conditions of ice morphology and therefore the final quality of frozen foods.

## Figures and Tables

**Table 1:** Optical microscopy applied to visualize ice crystals in different frozen food products.

Food Product	Microscopic Technique	Objectives	Reference
Large yellow croaker (*Pseudosciaena crocea*)	LM	Effects of ice crystals obtained through refrigerator (−20 °C), spiral freezer, and liquid nitrogen freezer, on water properties and protein stability	[35]
Snakehead (*Channa argus*)	LM	Effects of immersion freezing (−20, −30, and −40 °C) on ice crystal formation and protein properties in comparison to conventional air freezing (−20 °C)	[36]
Tilapia	LM	Obtention of equivalent diameter data of ice crystals to develop models for the prediction of ice crystal growth during recrystallization in frozen samples	[37]
Carrot	LM	Effect of direct current and alternating current magnetic field on the biological phase transformation and ice crystal formation	[38]
Tuna meat	LM	Effects of salting and subsequent freezing on the physicochemical and histological properties (diameter and area ratio of ice crystals)	[39]
Grass carp	LM	Effects of different freezing methods on ice crystals, distribution of water, and freshness properties during frozen storage	[40]
Coffee extract	LM	Effects of the application of annealing on the ice morphology, and the solute yield on block freeze concentrations	[41]
Common carp (*Cyprinus carpio*)	LM	Observation of ice crystals after ultrasound-assisted immersion freezing application	[42]
Microalgae Cells	Cryo-LM	Determine the most important parameters for the safety of cells in the process of cryopreservation: temperature parameters of phase transitions, the presence of extra- and intracellular ice, its structure, crystal size, and their growth rate	[43]
Fruits and vegetables (apple, peach, cucumber and Indian jujube)	Cryo-LM	Effects of static magnetic field (intensities between 0 to 45 mT) on micro and macroscales, comparing ice crystal size, time through −1 °C to −5 °C, drip loss and texture	[44]
Mushroom (*Agaricus bisporus*)	Cryo-LM	Observe the ice crystal morphology after the application of contact ultrasound (300 W, 20 kHz) during freezing and frozen storage	[45]
Mango Sorbet	Cryo-LM	Effect of ultrasound-assisted immersion freezing with respect to traditional freezing technologies	[46]
Honeydew melon	Cryo-LM	Observe the microstructure of samples exposed to CO_2_ pre-injection at different pressures combined with ultrasound-assistance (0.15 W/cm^2^ at 20 kHz) for quick freezing	[47]
Gel model food	Cryo-LM	Effects of freezing process assisted by electrostatic field, by static magnetic field, and by electrostatic field combined with static magnetic field on nucleation temperature of ice crystals	[48]

**Table 2:** Electron microscopy applied to visualize ice crystals in different frozen food products.

Food Product	Microscopic Technique	Objectives	Reference
Common carp (*Cyprinus carpio*)	TEM	Changes in primary, secondary, and tertiary structures of myofibril protein of frozen samples at different ultrasonic power level	[42]
Porcine longissimus muscle	TEM	Influence of multiple freeze-thaw cycles on (micro)structure damage, and myofibrillar proteins’ structure changes	[51]
Papaya tissues	TEM	Investigate the effect of freezing and thawing on texture, microstructure, and cell wall composition changes	[64]
Sliced Peaches	SEM	Determine if freezing rates and holding temperatures influence quality during short- and long-term frozen storage	[65]
Tomatoes	SEM	Effect of a combined osmo-dehydro-cryogenic-freezing process on quality characteristics of fruits and vegetables (lycopene content, color, and cell structures)	[66]
Red Radish	SEM	Effect of wrapped and ultrasonic treatment on freezing time, drip loss, texture, and sensory evaluation of red radish cylinders frozen by immersion freezing	[67]
Cherries	Cryo-SEM	Effects of the application of synthetic AFPs as a pretreatment in cherries before freezing to evaluate their potential to minimize freeze-thaw damage in frozen samples	[59]
Pork meet	Cryo-SEM	Identification of the degree of freeze damage in meat on analytic tool selection	[68]
Fresh sorbets	Cryo-SEM	Determine the effect of several stabilizers on the establishment of the microstructure	[69]
Grape tomato	Cryo-SEM	Evaluate the effects of isochoric freezing on microstructural changes after 4 weeks of preservation	[70]
Strawberry fruits	ESEM	Investigate the impacts of initial water content on the freezing and thawing profiles, the structural cell changes and textural characteristics, in fresh and partially dehydrated samples	[63]
Mango	ESEM	Relationship of vitamin C content with water state and ice crystals under different state/phase transitions (temperature fluctuations) during frozen storage	[71]
Cranberries	ESEM	Evaluate the effects of freezing and drying on the structure	[72]
Apple slices	ESEM	Evaluation of microstructures of the samples dried with novel non-thermal ultrasound contact drying method	[73]

**Table 3:** Non-destructive and non-invasive techniques applied to visualize ice crystals in different frozen food products.

Food Product	Microscopic Technique	Objectives	Reference
Pork patties	AFM	Explore the cryoprotective effect of ice structuring protein applied to myofibrillar protein during frozen storage	[87]
Pumpkin puree	AFM	Explored the mechanisms underlying the effects of pectin oligosaccharide on the quality control compared to trehalose and the changes in structure and properties during frozen storage	[88]
Dough	AFM	Effects of ultrasound-assisted freezing on the freezing time and water migration and the structural characteristics of gluten components	[89]
Surimi from Talang queenfish (*Scomberoides commersonnianus*)	AFM	Effects of active ice nucleation bacteria (*Pseudomonas syringae*) on freezing, ice crystal formation, aggregation, and oxidation of during frozen storage	[90]
Fish (*Trachurus murphyi)*	AFM	Investigate how freeze–thaw processing and storage affects myofibrillar protein	[91]
Noodles	AFM	Comprehensive theories for the strengthening effect of curdlan on quality from the perspective of gluten structure	[92]
Carrot	X-ray μCT	Visualization of 3D ice crystals during months with changes in temperature	[93]
Minced beef	X-ray μCT	Quantify 3D ice crystals in frozen samples according to the integral cooling rate (for mechanical and cryogenic freezing operating conditions)	[94]
Green asparagus	X-ray μCT	Influence of the various characteristics of ice crystals formed at different freezing rates on cell membrane damage and mechanical property	[95]
Strawberry	X-ray μCT	Effect of supercooled freezing on quality and the characteristics of ice crystals in frozen tissues prepared by supercooled freezing	[96]
Beef	NIR	Determine and assess quality parameters of meat product in frozen samples	[97]
Tuna	NIR	Discriminate between unfrozen and frozen-thawed samples	[98]
Pork	NIR	Monitoring the oxidative damage of myofibrils during frozen storage	[99]
Tilapia (*Oreochromis*)	NIR	Detect the frozen-thawed cycles in frozen fillets	[100]
Raw beef	Raman Spectroscopy	Predict the texture of different frozen/thaw samples from continuous freezing and repeated freeze-thaw treatments	[101]
Dough	Raman Spectroscopy	Effects of ultrasonic-assisted freezing on the water distribution and protein molecular structure	[102]
Sucrose solution	Raman Spectroscopy	Study the interactions between sucrose and water during freezing and explore the biophysical environment at interfaces between cells and nonfrozen sucrose solution, between cells and extracellular ice, and between nonfrozen sucrose solution and ice	[103]
Tamarind	Raman Spectroscopy	Evaluation of the interaction of seed polysaccharide with ice crystals and water molecules with a view to gaining a greater understanding of the action mechanism.	[104]
Largemouth bass fish (*Micropterus salmoides*)	Raman Spectroscopy	Study the quality during freezing with pressure changes in storage at −30 °C in comparison to two freezing techniques	[105]
Cod (*Gadus morhua*)	MRI	Map and quantify tissue damage from freezing	[106]
Korla fragrant pear	NMR MRI	Explore the influence of different static magnetic field on the product quality	[107]
Pork tenderloin	NMR	Effect of different freezing methods on the moisture state and moisture distribution of thawed pork tenderloin	[108]
Noodles	NMR MRI	Determine the water distribution and migration during frozen storage for 12 weeks	[109]

## Data Availability

Not applicable.
